# Serotonin transporter inhibition and 5-HT_2C_ receptor activation drive loss of cocaine-induced locomotor activation in DAT Val559 mice

**DOI:** 10.1038/s41386-018-0301-8

**Published:** 2018-12-21

**Authors:** Adele Stewart, Gwynne L. Davis, Paul J. Gresch, Rania M. Katamish, Rodeania Peart, Maximilian J. Rabil, Raajaram Gowrishankar, F. Ivy Carroll, Maureen K. Hahn, Randy D. Blakely

**Affiliations:** 1 Department of Biomedical Science, Florida Atlantic University, Jupiter, FL USA; 2 Neuroscience Graduate Program, Vanderbilt University, Nashville, TN USA; 3 Brain Institute, Florida Atlantic University, Jupiter, FL USA; 4 Wilkes Honors College, Florida Atlantic University, Jupiter, FL USA; 5 International Scholars Program, Vanderbilt University, Nashville, TN USA; 6 Research Triangle Institute, Research Triangle Park, NC USA

**Keywords:** Transporters in the nervous system, ADHD

## Abstract

Dopamine (DA) signaling dysfunction is believed to contribute to multiple neuropsychiatric disorders including attention-deficit/hyperactivity disorder (ADHD). The rare DA transporter (DAT) coding substitution Ala559Val found in subjects with ADHD, bipolar disorder and autism, promotes anomalous DA efflux in vitro and, in DAT Val559 mice, leads to increased reactivity to imminent handling, waiting impulsivity, and enhanced motivation for reward. Here, we report that, in contrast to amphetamine and methylphenidate, which induce significant locomotor activation, cocaine administration to these mice elicits no locomotor effects, despite retention of conditioned place preference (CPP). Additionally, cocaine fails to elevate extracellular DA. Given that amphetamine and methylphenidate, unlike cocaine, lack high-affinity interactions with the serotonin (5-HT) transporter (SERT), we hypothesized that the lack of cocaine-induced hyperlocomotion in DAT Val559 mice arises from SERT blockade and augmented 5-HT signaling relative to cocaine actions on wildtype animals. Consistent with this idea, the SERT blocker fluoxetine abolished methylphenidate-induced locomotor activity in DAT Val559 mice, mimicking the effects seen with cocaine. Additionally, a cocaine analog (RTI-113) with greater selectivity for DAT over SERT retains locomotor activation in DAT Val559 mice. Furthermore, genetic elimination of high-affinity cocaine interactions at SERT in DAT Val559 mice, or specific inhibition of 5-HT_2C_ receptors in these animals, restored cocaine-induced locomotion, but did not restore cocaine-induced elevations of extracellular DA. Our findings reveal a significant serotonergic plasticity arising in the DAT Val559 model that involves enhanced 5-HT_2C_ signaling, acting independently of striatal DA release, capable of suppressing the activity of cocaine-sensitive motor circuits.

## Introduction

The release of dopamine (DA) at synapses located in the dorsal striatum, nucleus accumbens, and prefrontal cortex sustains complex behaviors including locomotion, reward, motivation, attention, and executive function. Pathological alterations in DA homeostasis and signaling have been implicated in multiple neuropsychiatric disorders including bipolar disorder (BPD), schizophrenia, attention-deficit/hyperactivity disorder (ADHD), and substance abuse disorders. Although significant evidence exists of disrupted DA signaling in these disorders, the mechanisms by which these changes arise, and the impact that these insults have on synaptic and circuit plasticities, remains an active area of investigation.

The presynaptic DA transporter (DAT, *SLC6A3*) is a major determinant of DA clearance and inactivation of DA signaling [1]. Drugs used in the treatment of ADHD such as amphetamine and methylphenidate, as well as the powerful psychostimulant cocaine, target DAT, though they alter transporter function through distinct mechanisms. Both methylphenidate and cocaine are DAT inhibitors that elicit rapid elevations in extracellular DA following vesicular DA release. In contrast, amphetamine acts as a competitive DAT substrate and, after intracellular accumulation, can bias DAT toward a DA efflux-prone conformation [2, 3], resulting in transporter-mediated DA release. Cocaine, unlike amphetamine and methylphenidate, exhibits a high affinity interaction with the serotonin (5-HT) transporter (SERT) [4]. The DA-linked behavioral traits of ADHD (i.e., hyperactivity, impulsivity, inattention) and the ability of DAT-targeted drugs to treat ADHD have compelled hypotheses that changes in DAT expression or function might underlie risk for the disorder. Though findings from positron emission tomography (PET) studies that have sought to associate alterations in DAT brain levels to ADHD are mixed [5, 6], genetic studies have consistently reported a link between variable nucleotide tandem repeats of the *Slc6a3* gene and the disorder [7–9].

The evaluation of common genetic variation permits population level assessment of the role of DAT in disease risk. However, the variants monitored in such studies are often not conserved in animals where mechanistic studies can be implemented. To overcome this issue, we and others have pursued the identification of rare coding variation that impact DAT function [10–13]. The DAT Val559 variant has, to date, been identified in two adolescent male siblings with ADHD [14], a female with BPD [15], and two unrelated boys with autism spectrum disorder (ASD) [16], though it has also been found in unaffected individuals. In a heterologous expression system, DAT Val559 transfected cells conferred total and surface protein levels and DA uptake equivalent to levels obtained with wildtype DAT, and the ability of psychostimulants to inhibit DA uptake demonstrated no impact of the mutant allele [10]. However, amperometric measurements coupled with whole cell patch-clamp approaches revealed DAT Val559 to support a spontaneous outward DA leak [17]. Moreover, in contrast to the ability of amphetamine to elicit DA efflux in cells transfected with wildtype DAT, amphetamine suppresses spontaneous DA efflux in cells expressing the DAT Val559 variant, mimicking the effects seen with methylphenidate.

In an effort to test the impact of DAT Val559 on DA homeostasis in vivo, our group generated DAT Val559 knock-in mice [18, 19]. Though genetic elimination of DAT results in profound hyperactivity [20], we found the DAT Val559 variant to produce more subtle alterations in spontaneous locomotion, specifically a hyperactive locomotor response to imminent handling (“darting”). Additionally, mutant mice displayed blunted (though detectible), locomotor responses to amphetamine and methylphenidate [19]. Additionally, cognitive testing of DAT Val559 mice revealed a waiting impulsivity that appears to be driven by a heightened reward motivation [21]. Ex vivo DA release studies using striatal slices, along with in vivo microdialysis and chronoamperometry experiments [19, 22] reported tonic DA efflux supported by constitutive activation of presynaptic DA D2 autoreceptors in DAT Val559 mice. Tonic activation of D2 autoreceptors was also recently reported to drive elevated DAT Val559 surface expression ex vivo, amplifying non-vesicular DA release specifically in the dorsal striatum [22].

Given our findings of increased reward motivation in the DAT Val559 mice, we extended our study of psychostimulant actions in these mice from the therapeutic agents, amphetamine and methylphenidate, to a psychostimulant that is commonly abused, cocaine. Below we report that DAT Val559 mice demonstrate a striking locomotor insensitivity to cocaine in the context of normal cocaine reward, as revealed through conditioned place preference (CPP) studies. We establish that the locomotor insensitivity to cocaine of DAT Val559 mice reflects the emergence of a 5-HT_2C_ receptor-supported serotonergic plasticity that suppresses locomotion through mechanisms independent of the DAT Val559 allele’s ability to suppress striatal dopamine release.

## Materials and methods

### Mice

Wildtype and homozygous DAT Val559 littermate mice were bred from heterozygous breeders maintained on a hybrid background (75% 129S6/SvEvTac and 25% C57BL/6J) [19]. Double mutant SERT Met172 and DAT Val559 mice were generated by crossing heterozygous DAT Val559 mice with homozygous SERT Met172 mice on a pure 129S6 background. After the first filial (F1) generation two distinct lines were maintained where breeders were heterozygous for the DAT Val559 allele and homozygous for either the wildtype or SERT Met172 allele. All experiments utilized male mice. Mice were housed on a 12:12 light/dark cycle. For the majority of experiments, mice were bred on a standard light cycle (lights on/off at 7 am and 7 pm, respectively) and transferred to reverse light cycle housing (lights on/off at 3 am and 3 pm, respectively) at 5 weeks of age. A subset of data (Figs. 5, 6, and S6) was generated from mice bred, raised, and continually housed on a reverse light cycle. Each cohort consisted of approximately equal numbers of wildtype and DAT Val559 mice such that the datasets are evenly distributed across genotypes. We have no evidence that the differences in housing impacted drug or genotype effects. As ADHD symptomology tends to manifest during adolescence in human subjects, behavioral testing was performed on mice between 6 and 9 weeks of age. Due to technical limitations, microdialysis experiments utilized 10–12 weeks old mice. All experiments utilizing mice were performed under a protocol approved by the Institutional Animal Care and Use Committee (IACUC) at either Vanderbilt University or Florida Atlantic University.

### Behavioral assays

Behavioral testing was completed in either the Laboratory for Neurobehavior Core Facility operated by the Vanderbilt Brain Institute or the Neurobehavior Core Facility under the auspices of the Florida Atlantic University Brain Institute. Experiments were performed in either facility during the active (dark) phase of the light/dark cycle under red light. Wherever possible, testing was confined to between 6 am and 12 pm to avoid the confounding influence of diurnal variation in extracellular DA tone [23]. Mice were habituated to the testing rooms for a minimum of 20 min prior to the start of each experiment. All behavioral assays were performed by an experimenter blinded to animal genotype. For all experiments, data are combined from at least two independent animal cohorts.

### Locomotor activity testing

Drug-induced hyperlocomotion was measured using Med Associates activity chambers as previously described [19]. For the majority of experiments, activity testing was performed with prior habituation wherein, on day 1, mice were placed in the chamber for 30 min to acclimate to the testing environment. Two days later, mice were again habituated for 30 min, injected with sterile saline (0.9% NaCl) or 0.01% dimethyl sulfoxide (DMSO) in saline (fluoxetine only) and activity was recorded for 60 or 120 (RTI-113 only) min post-injection. On the final day of testing, mice received drug injections following an initial 30 min habitation and activity was again monitored for 60 or 120 (RTI-113 only) min post-injection. Habituation was omitted for all testing with DAT Val559/SERT Met172 hybrid mice and their littermate controls, as well as for experiments with SDZ SER-082, a 5-HT_2C_ receptor (5-HT_2C_R) antagonist. For studies with SDZ SER-082, the drug or saline was administered 30 min prior to cocaine injection and locomotor testing. The following drugs were obtained from the vendors noted, dissolved in sterile saline and administered as noted via intraperitoneal injection (i.p.): cocaine HCl, Sigma, St. Louis, MO (10, 30 mg/kg); methylphenidate HCl, Sigma (10 mg/kg); RTI-113 (2β-carbophenoxy-3β-(4-chlorophenyl)tropane), Research Triangle Institute, Research Triangle Park, NC (2 mg/kg); SDZ SER-082 fumarate, Tocris, Minneapolis, MN (0.5 mg/kg). Fluoxetine HCl was purchased from Sigma and dissolved in saline with 0.01% DMSO at 20 mg/kg.

### Conditioned place preference

CPP for cocaine was established in wildtype and DAT Val559 mice utilizing a biased design previously used to evaluate cocaine CPP in DAT knockout mice [24]. The apparatus consisted of an insert dividing locomotor chambers into two equally sized areas with distinctive tactile cues (mesh vs grid rod flooring). Initial preference was established by allowing mice access to both sides of the chamber for 20 min. A biased approach was used to assign the cocaine (CS+) or saline (CS−) paired sides of the chamber to avoid introducing a false positive genotype effect as all wildtype mice exhibited a pronounced initial bias for the mesh flooring whereas approximately 40% of DAT Val559 mice preferred the rod floor. Thus, CS+ was assigned to the mice’s least preferred side on an individual animal basis. For the next 8 days, mice were subject to alternating saline or cocaine (10 mg/kg, i.p.) injections followed by placement on the CS− or CS+ side of the chamber, respectively, for 20 min. On day 10, chamber side preference was reassessed by raising the dividing door, placing mice on the CS− side, and allowing mice access to both sides of the chamber for 20 min. Cocaine CPP was then extinguished through a series of 3 paired extinction trials where mice were injected with saline and placed on the CS+ or CS− side on alternating days followed by a preference test every third day.

### Microdialysis

Microdialysate was collected from awake, ambulatory wildtype, and DAT Val559 mice following a single cocaine injection (10 mg/kg, i.p.). All surgeries and sample collection were performed during the inactive phase. Probes were surgically implanted according to our previously published protocol [19] with the guide cannula positioned in the dorsal striatum (−0.86 AP from bregma, ±1.6 ML, and −2.0 DV from dura; Figure S5). Approximately 18–24 h after surgery samples were collected every 20 min including 4 baseline samples and an additional 6 following intraperitoneal injection of cocaine (10 mg/kg, i.p.). Dialysate samples were stored at −80 °C and analyzed by HPLC-EC for DA levels as described previously [19].

### Synaptosomal [^3^H]DA and [^3^H]5-HT uptake inhibition

Synaptosomes were prepared from the striata of wildtype, DAT Val559 and SERT Met172 mice and [^3^H]DA and [^3^H]5-HT uptake assays performed for 10 min at 37 °C as previously described [19, 25]. Synaptosomes were pre-incubated with drugs [cocaine, 10^−9^–10^−3^ M; RTI-113, 10^−9^–10^−4^ M] for 10 min at 37 °C prior to the addition of 50 nM [^3^H]5-HT (specific activity 28 Ci/mmol; PerkinElmer, Waltham, MA) or 50 nM [^3^H]DA (specific activity 46 Ci/mmol; PerkinElmer). Control samples were incubated with assay buffer only, and non-specific uptake was determined in parallel samples to which 1 μM GBR-12909 (Sigma) or paroxetine (Sigma) were added to inhibit DAT or SERT, respectively. Assays were performed in triplicate for all conditions, averaged, and expressed relative to control.

### Quantitation of tissue cocaine content

Wildtype and DAT Val559 mice were given a single cocaine injection (10 mg/kg, i.p.), euthanized via rapid decapitation after 10 min and the whole striatum rapidly dissected on a pre-chilled metal platform. Tissue samples were flash frozen and stored at −80 °C until processed. Cocaine concentrations in striatal tissue were determined via liquid chromatography/tandem mass spectrometry at the Drug Metabolism & Pharmacokinetics Core Facility at the Scripps Research Institute (Jupiter, FL), overseen by Michael D. Cameron, Ph.D.

### SERT immunoblotting experiments

Brains were harvested from mice following rapid decapitation, placed on a pre-chilled metal platform, and the striata quickly dissected. Tissue samples were homogenized in RIPA buffer (150 mM NaCl, 50 mM Tris–HCl, 2 mM EDTA, 1% Triton-X, 0.1% SDS, 1% sodium deoxycholate) with protease inhibitor cocktail (Sigma). Samples were vortexed and centrifuged at 14,000×*g* for 10 min. 30 μg protein was resolved by SDS-PAGE. Following transfer to PVDF membranes, SERT was detected with a guinea pig anti-SERT primary antibody (1:2000; 5-HTT-GP-Af1400, RRID: AB_2571777, Frontier Institute, Japan) and HRP-conjugated rabbit anti-guinea pig secondary antibody (1:10,000, A-5545, RRID: AB_258247, Sigma). Western blots were visualized and quantitated by enhanced chemiluminescence (BioRad Clarity ECL, Hercules, CA) using an ImageQuant LAS 4000 imager (GE Healthcare Life Sciences, Chicago, IL). Actin served as a loading control (1:10,000, A5541, Sigma).

### Statistical analysis

Data are presented as mean ± SEM and were analyzed using Prism 6.0 (GraphPad Inc, La Jolla, CA). Statistical significance was set at *P* < 0.05 for all analyses. A Student’s unpaired, two-sided *t*-test was used to compare between two independent data sets. One-way analysis of variance (ANOVA) with Dunnett’s post-hoc test was utilized for experiments with a single independent variable and more than 2 groups. For data containing two independent variables, two-way ANOVA was performed followed by Tukey’s multiple comparison test (4 groups) or Sidak’s post-hoc test (>4 groups). Repeated measures ANOVA (rmANOVA) was utilized with Sidak’s post-hoc tests for time course data sets. Uptake inhibition best-fit curves were generated using the log(inhibitor) vs response standard curve function in Prism, allowing for variable slope.

## Results

### Cocaine fails to elicit hyperlocomotion, stereotypy, or rearing in DAT Val559 mice

**Fig. 1:**
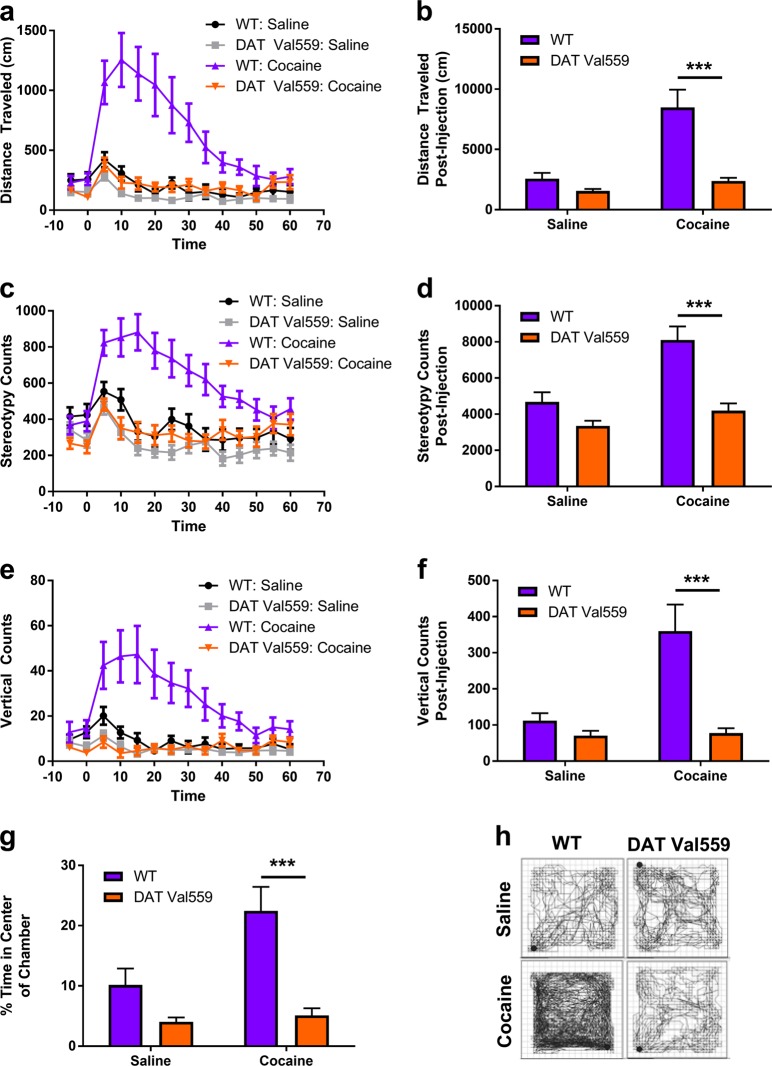
Cocaine-induced locomotion is absent in DAT Val559 mice. Wildtype (WT, *n* = 13) and homozygous DAT Val559 (*n* = 19) mice were injected with saline or cocaine (10 mg/kg, i.p.) and locomotor activity measured for 60 min post-injection. **a**, **b** Distance traveled, **c**, **d** stereotypy counts, and **e**, **f** vertical counts are depicted in 5 min bins or summarized as post-injections totals. **g** Cocaine increased the time spent in the center of the chamber in WT but not DAT Val559 mice. **h** Representative activity traces. ***P < 0.001 via Tukey’s post-hoc test. Data are presented as mean ± SEM

We began our investigation into the behavioral impact of cocaine in DAT Val559 mice by assessing cocaine-induced hyperactivity. In contrast to results obtained with amphetamine and methylphenidate [19], cocaine failed to produce locomotor activation in DAT Val559 mice (Fig. 1a, b), nor did the drug promote stereotypic activity (Fig. 1c, d) or rearing (Fig. 1e, f). Two-way ANOVA revealed a significant effect of drug and genotype for total distance traveled [*F*(1,55) = 22.54, *P* < 0.0001; *F*(1,55) = 25.10, *P* < 0.0001], stereotypy counts [*F*(1,55) = 18.80, *P* < 0.0001; *F*(1,55) = 28.24, *P* < 0.0001], and vertical counts [*F*(1,55) = 12.92, *P* = 0.0007; *F*(1,55) = 20.87, *P* < 0.0001]. In addition, though cocaine promoted center occupancy indicative of anxiolysis in wildtype mice, similar effects were not seen in DAT Val559 mice (Fig. 1g, h) [Two-way ANOVA: drug, *F*(1,55) = 9.21, *P* = 0.0037; genotype, *F*(1,55) = 28.35, *P* < 0.0001]. Even at high doses (30 mg/kg, i.p.) cocaine failed to elicit hyperlocomotion in DAT Val559 mice (Figure S1a-c), though, at this dose, we observed no increase in stereotypy (Figure S1d-e) or rearing (Figure S1f-g) in mice of either genotype. Two-way ANOVA revealed a significant effect of drug and genotype for total distance traveled only [drug, *F*(1,60) = 13.63, *P* = 0.0005; genotype, *F*(1,60) = 5.051, *P* = 0.0283]. Together, these data indicate that the efficacy of cocaine in triggering motor activation is significantly compromised in DAT Val559 mice.

### Cocaine-mediated inhibition of ex vivo DA uptake and in vivo cocaine accumulation in brain are unchanged in DAT Val559 mice

**Fig. 2:**
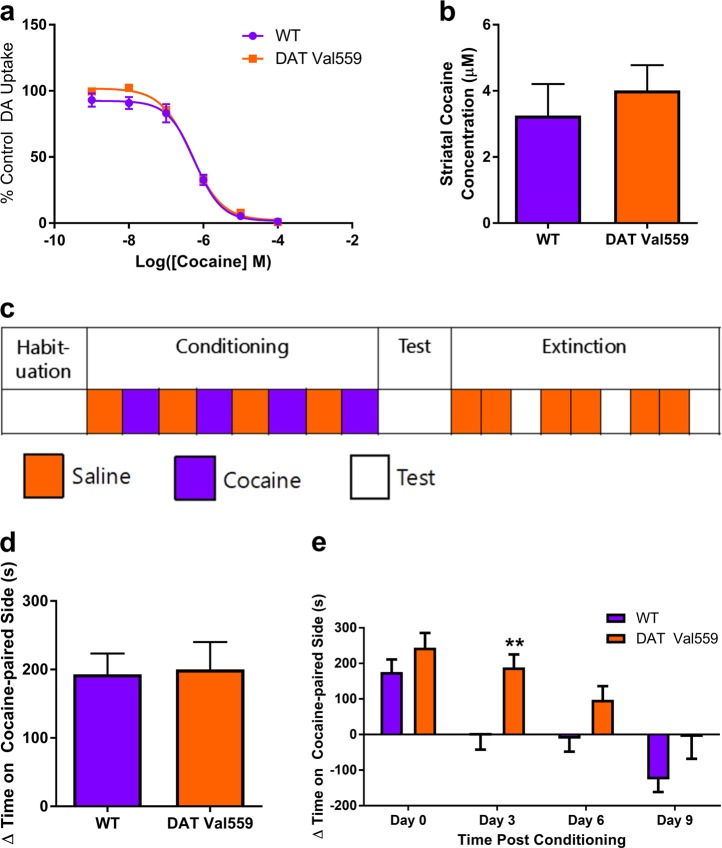
Cocaine-mediated inhibition of DA uptake, brain accumulation, and conditioned place preference remain intact in DAT Val559 mice. **a** Inhibition of specific DA uptake was assessed in striatal synaptosomes isolated from wildtype (WT) and DAT Val559 mice (*n* = 6) exposed to increasing concentrations of cocaine (10^−9^ to 10^−4^ M). Nonlinear curves were fit to the data to determine IC_50_ values (WT, 583 ± 117 nM; DAT Val559, 473 ± 48.3 nM). **b** Cocaine content in striatal tissue isolated from WT and DAT Val559 mice (*n* = 6) 10 min after cocaine injection (10 mg/kg, i.p.). Tissue cocaine content (ng cocaine/g tissue) was converted to µM by assuming 1 g brain tissue is equivalent to 1 ml final volume. **c** WT (*n* = 16) and DAT Val559 (*n* = 17) mice underwent cocaine place preference conditioning according to the outlined protocol. **d** Change (∆) in time spent on the cocaine-paired side (CS+) of the testing apparatus following the conditioning phase (4 pairs of saline or cocaine injections). **e** ∆Time spent on the cocaine-paired side (CS+) of the testing apparatus for each of 3 extinction phase testing days. **P < 0.01 vs WT via Sidak’s post-hoc test. Data are presented as mean ± SEM

Several potential mechanisms could explain the loss of cocaine-induced locomotion in DAT Val559 mice, foremost being a loss of cocaine potency for DA uptake inhibition. Our previous studies demonstrated that the ability of cocaine to inhibit DAT-dependent DA uptake was unchanged in cells expressing the human DAT Val559 variant [10], results we replicate here using striatal synaptosomes isolated from DAT Val559 mice (IC_50_ 583 ± 117 nM in wildtype mice vs 473 ± 48.3 nM in DAT Val559, Fig. 2a). Another explanation for a loss of cocaine action could be a mutation-induced change in cocaine levels achieved in the brain following peripheral injection. However, cocaine levels in the striatum of DAT Val559 mice 10 min after an i.p. cocaine injection (10 mg/kg) were statistically equivalent to levels found in wildtype mice (Fig. 2b).

### DAT Val559 mice exhibit normal cocaine CPP acquisition with delayed extinction

Given the complete loss of a psychomotor response to cocaine in DAT Val559 mice, we next sought to test another behavioral dimension of cocaine action: reward. To this end, wildtype and DAT Val559 mice underwent cocaine place preference conditioning (Fig. 2c). As expected, wildtype mice demonstrated a preference for the cocaine-paired side of the test chamber following conditioning (Fig. 2d). In contrast to locomotor findings, DAT Val559 mice exhibited CPP for cocaine at levels equivalent to wildtype mice (Fig. 2d). However, whereas wildtype mice lost cocaine CPP by day 3 post-conditioning, DAT Val559 mice failed to extinguish cocaine CPP until day 9 (Fig. 2e). Two-way ANOVA revealed a significant effect of testing day (*F*(3,109) = 27.02, *P* < 0.0001) and genotype (*F*(1,109) = 9.813, *P* < 0.0001).

### DAT Val559 mice demonstrate SSRI-induced antagonism of methylphenidate-induced locomotion

**Fig. 3:**
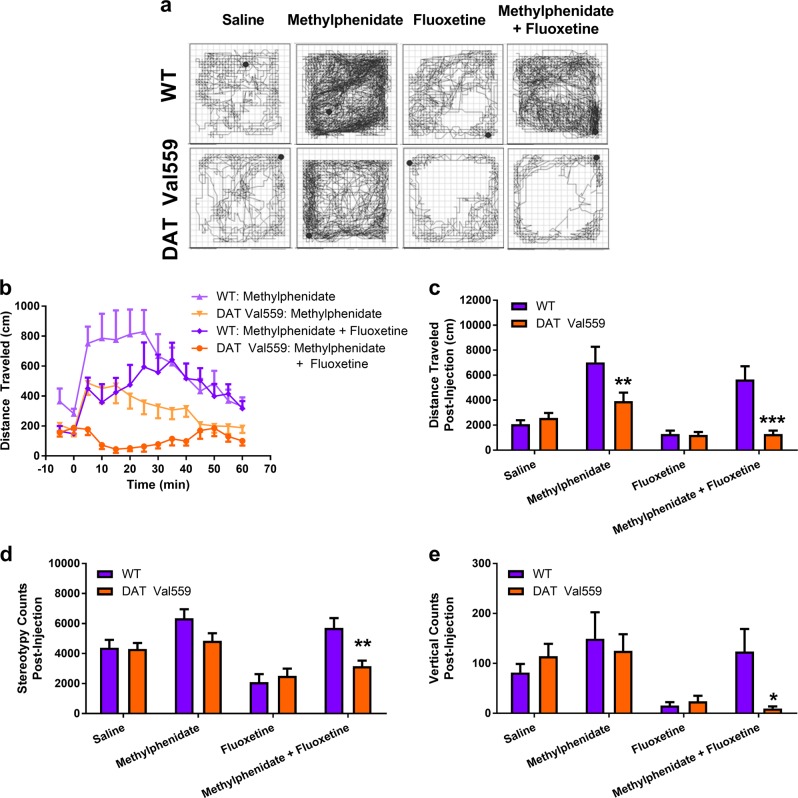
DAT Val559 mice are sensitized to the 5-HT-driven inhibition of DA-mediated locomotor behavior. **a** Representative activity traces from wildtype (WT, *n* = 12–14) and DAT Val559 (*n* = 10–15) mice given a single injection containing saline, methylphenidate (10 mg/kg, i.p.), fluoxetine (20 mg/kg, i.p.) or co-injected with both methylphenidate and fluoxetine. **b** Distance traveled for 60 min post-injection in 5 min bins for methylphenidate and methylphenidate + fluoxetine injected WT and DAT Val559 mice. **c** Cumulative distance traveled post-injection. **d** Cumulative stereotypy counts post-injection. **e** Cumulative vertical counts post-injection. *P < 0.05, **P < 0.01, ***P < 0.001 vs WT via Sidak’s post-hoc test. Data are presented as mean ± SEM

The inability of cocaine to trigger locomotor activation in DAT Val559 mice was puzzling, due to intact, though blunted, locomotor stimulation by methylphenidate, a drug that also inhibits DAT [19]. However, a major difference between cocaine and methylphenidate is a high-affinity interaction with the 5-HT transporter (SERT) [4], suggesting that serotonergic mechanisms might underlie the loss of locomotor stimulation by cocaine in the mutant mice. If this is the case, we reasoned that treatment of DAT Val559 mice with a 5-HT selective reuptake inhibitor (SSRI) should suppress methylphenidate-induced locomotor activation in DAT Val559 mice. We therefore treated wildtype and DAT Val559 concurrently with methylphenidate and the SSRI fluoxetine (20 mg/kg i.p.). As we previously reported, methylphenidate induced locomotor activation in DAT Val559 mice, though at a reduced level compared to wildtype mice (Fig. 3a, b). Fluoxetine caused a small, transient, but non-significant dip in activity in both wildtype and DAT Val559 mice (Figure S2). However, although fluoxetine had no significant effect on the total distance traveled in methylphenidate-treated wildtype mice (Fig. 3b), it completely abolished methylphenidate-induced hyperactivity in DAT Val559 mice (Fig. 3c) [Two-way ANOVA revealed a significant effect of drug (*F*(3,90) = 13.89, *P* < 0.0001) and genotype (*F*(1,90) = 12.93, *P* = 0.0005)]. Further, fluoxetine blunted methylphenidate-induced stereotypy and vertical exploratory behavior in DAT Val559 but not wildtype mice (Fig. 3d, e). Two-way ANOVA revealed a significant effect of drug (*F*(3,90) = 12.50, *P* < 0.0001) and genotype (*F*(3,90) = 6.237, *P* = 0.0143) on cumulative stereotypy counts post-injection and drug (*F*(3.90) = 4.899, *P* = 0.0034) on cumulative vertical counts post-injection. As striatal levels of SERT protein were equivalent between wildtype and DAT Val559 mice (Figure S3a-b), the consequences of SERT blockade by fluoxetine, rather than the capacity for SERT inhibition, likely underlie the emergence of SSRI inhibition of methylphenidate-induced motor activation.

### A cocaine analog with increased DAT vs SERT selectivity triggers locomotor activation in DAT Val559 mice

**Fig. 4:**
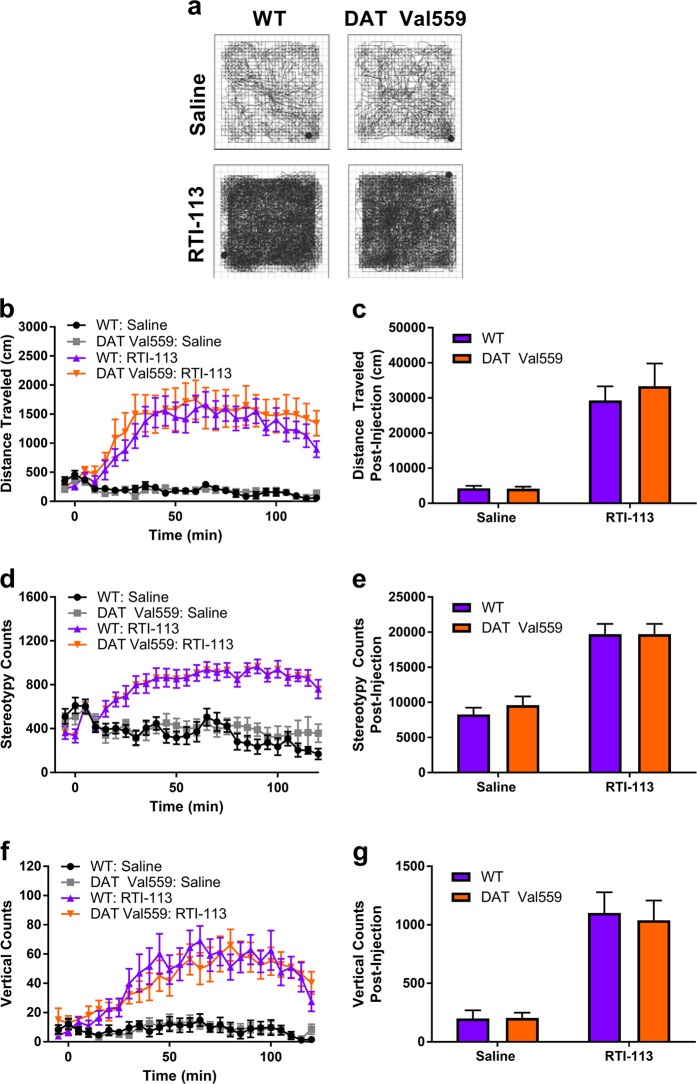
Hyperlocomotion in response to the DAT-selective cocaine analog RTI-113 is unchanged in DAT Val559 mice. **a** Representative activity traces from wildtype (WT, *n* = 10) and DAT Val559 (*n* = 11) mice injected with RTI-113 (2 mg/kg, i.p.). **b**, **c** Distance traveled, **d**, **e** stereotypy counts, and **f**, **g** vertical counts post-injection are depicted in 5 min bins or summed over the 2 h recording period. Data are presented as mean ± SEM

One caveat to experiments described in Fig. 3 is the observed, non-significant, and seeming transient suppression of methylphenidate-induced locomotor activity in wildtype mice by fluoxetine, possibly a result of off-target inhibition of 5-HT receptors [26]. To diminish this possibility, we investigated the response of DAT Val559 mice to a cocaine analog engineered with improved DAT/SERT selectivity. Whereas cocaine exhibits approximately equivalent affinity for the monoamine transporters (IC_50_ mouse DAT, 0.49 μM; NET, 0.46 μM; SERT, 0.74 μM) [4] in mouse striatal synaptosomes, RTI-113 exhibits significantly higher potency for DAT inhibition (wildtype, IC_50_ = 74.1 ± 5.5 nM; DAT Val559, IC_50_ = 47.8 ± 14.3 nM) compared to SERT (wildtype, IC_50_ = 4.15 ± 2.05 μM; DAT Val559, IC_50_ = 4.93 ± 1.77 μM) (Figure S4). Thus, if hyper-responsiveness to 5-HT contributes to loss of cocaine-induced locomotion in DAT Val559 mice, we would expect the increased DAT selectivity of RTI-113 to induce locomotor activation more similar to amphetamine or methylphenidate [19]. Indeed, at 2 mg/kg, i.p., RTI-113 increased horizontal (Fig. 4a–c) and vertical (Fig. 4f, g) locomotor activity as well as stereotypy (Fig. 4d, e) comparably between wildtype and DAT Val559 mice. Two-way ANOVA showed a significant effect of drug only for distance traveled (*F*(1,42) = 51.97, *P* < 0.0001), stereotypy (*F*(1,42) = 70.59, *P* < 0.0001), and rearing (*F*(1,42) = 48.69, *P* < 0.0001).

### Genetic elimination of high-affinity cocaine recognition by SERT restores cocaine-induced hyperlocomotion in DAT Val559 mice

**Fig. 5:**
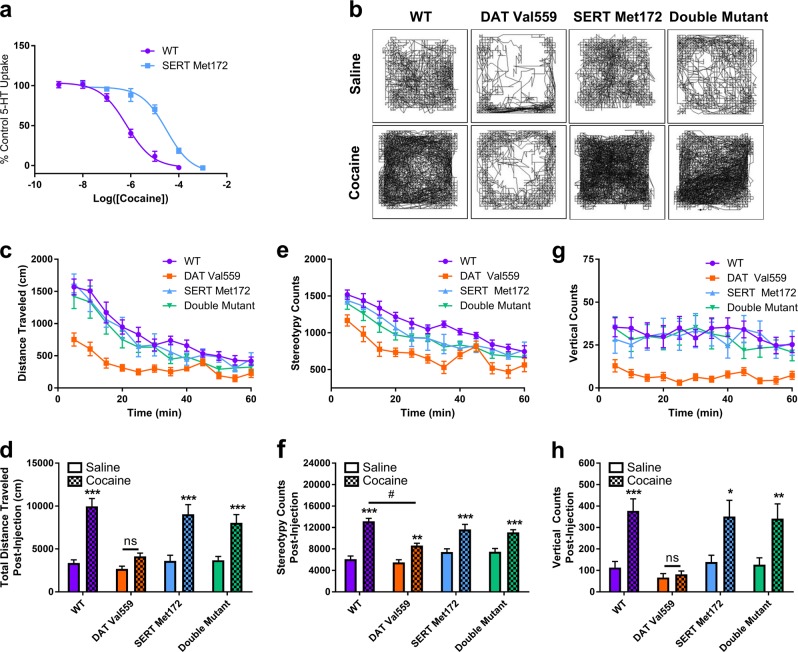
Genetic elimination of SERT’s cocaine binding capacity restores cocaine-induced locomotion in DAT Val559 mice. **a** Inhibition of specific 5-HT uptake was assessed in striatal synaptosomes isolated from wildtype (WT) and SERT Met172 mice (*n* = 5) exposed to increasing concentrations of cocaine (10^−9^ to 10^−3^ M). Nonlinear curves were fit to the data to determine IC_50_ values (WT, 670 ± 180 nM; SERT Met172, 30.7 ± 9.18 μM). **b** Representative activity traces of WT (*n* = 13), DAT Val559 (*n* = 11), SERT Met172 (*n* = 8), and double mutant (*n* = 10) mice given an injection of saline or cocaine (10 mg/kg, i.p.). **c**, **d** Distance traveled, **e**, **f** stereotypy counts, and **g**, **h** vertical counts post-injection are depicted in 5 min bins or summed over the 60 min recording period. *P < 0.05, **P < 0.01, ***P < 0.001 vs saline-treated control via Sidak’s post-hoc test. ^#^*P* < 0.001 vs cocaine treated WT mice. ns = not significantly different. Data are presented as mean ± SEM

To confirm a role for SERT blockade in the anomalous loss of cocaine locomotor activation in the DAT Val559 mice, we capitalized on the ability of the SERT Met172 mutation to retain normal SERT function but significantly reduce cocaine affinity at SERT, findings documented in vitro [27] and in vivo using SERT Met172 knock-in mice [28]. Importantly, our prior work revealed that the SERT Met172 mutation fails to impact SERT protein expression, 5-HT transport or 5-HT levels in vivo [25]. Consistent with prior findings of reduced cocaine potency with SERT Met172 expressed on a pure C57BL/6 background [25], cocaine potency for SERT-dependent, striatal 5-HT uptake inhibition was reduced by 45-fold when the SERT Met172 mutation was placed on the hybrid 129S6-C57BL/6 background used in our DAT Val559 studies (Fig. 5a). Importantly, when the SERT Met172 mutation was expressed together with the DAT Val559 variant, cocaine locomotor activation (Fig. 5b–d), and rearing (Fig. 5g, h), completely absent in DAT Val559 mice, and stereotypy (Fig. 5e, f), blunted compared to wildtype mice, were restored. The discrepancy in cocaine-dependent stereotypic behaviors between data presented in Fig. 1 and this experiment may derive from the different genetic backgrounds used, the confounding influence of novelty resulting from a lack of prior habituation to the testing apparatus, or an unknown environmental variable. Regardless, two-way ANOVA showed a significant effect of drug and genotype for distance traveled (*F*(1,76) = 82.61, *P* < 0.0001; *F*(3,76) = 9.84, *P* < 0.0001), stereotypy (*F*(1,76) = 112.9, *P* < 0.0001; *F*(3,76) = 8.78, *P* < 0.0001), and rearing (*F*(1,76) = 30.2, *P* < 0.0001; *F*(3,76) = 7.5, *P* = 0.0002). Overall, these data provide strong support for the hypothesis that the failure of cocaine to induce open field locomotor activation in DAT Val559 mice arises from plasticity induced by the anomalous DA efflux of DAT Val559 that sensitizes serotonergic inhibitory drive on the motor circuit.

### Lack of effect of DAT Val559 on striatal 5-HT elevations following cocaine administration

**Fig. 6:**
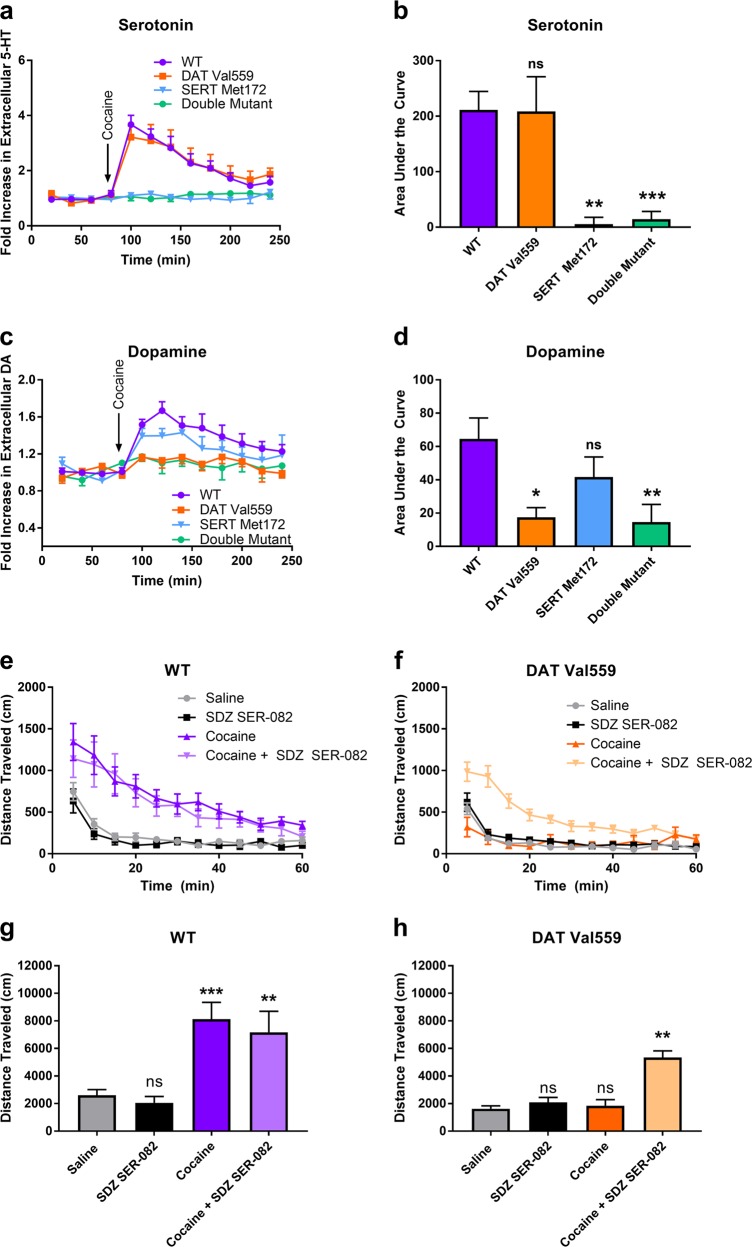
Blockade of 5-HT_2C_ receptor signaling restores cocaine-induced locomotion in DAT Val559 mice independent of DA release. Wildtype (WT, *n* = 6–7), DAT Val559 (*n* = 5–6), SERT Met172 and DAT Val559/SERT Met172 hybrids (double mutant, *n* = 7) were injected with cocaine (10 mg/kg, i.p.) and **a**, **b** 5-HT and **c**, **d** DA elevations in the striatum measured using in vivo microdialysis. Eluates were pooled in 20 min bins and expressed as averages over time (left) or area under the curve (right). Average baseline DA concentration was 3.22 ± 0.60 nM (WT) and 1.56 ± 0.34 nM (DAT Val559). Average baseline 5-HT concentration was 0.082 ± 0.017 nM (WT) and 0.057 ± 0.011 nM (DAT Val559). Total distance traveled in **e**, **g** WT (*n* = 10–12) and **f**, **h** DAT Val559 (*n* = 10–11) mice injected with saline or cocaine (10 mg/kg, i.p.) following a 30 min pre-exposure to saline or SDZ SER-082 (0.5 mg/kg, i.p.). Data are presented in 5 min bins or summed over the 60 min recording period. *P < 0.05, **P < 0.01, ***P < 0.001 vs saline-treated control via Sidak’s or Dunnett’s post-hoc test. ^#^*P* < 0.001 vs cocaine treated WT mice. ns = not significantly different. Data are presented as mean ± SEM

A greater increase in extracellular 5-HT following SERT blockade by cocaine in DAT Val559 mice, as compared to wildtype animals, could underlie the emergence of anomalous, SERT-mediated suppression of cocaine’s locomotor effects. Alternatively, loss of locomotor activation could derive from increased signaling by postsynaptic 5-HT receptors. As shown in Fig. 6a, b, microdialysis studies in the striatum (Figure S5) revealed an equivalent elevation of extracellular 5-HT in both wildtype and DAT Val559 mice after i.p., cocaine (10 mg/kg) administration, and as such changes in extracellular 5-HT resulting from SERT antagonism in this region cannot account for a loss of cocaine-induced locomotion in DAT Val559 mice. As expected, SERT Met172 mice, and DAT Val559 mice expressing the SERT Met172 mutation, failed to exhibit 5-HT elevations (Fig. 6a, b) [One-way ANOVA: *F*(3,19) = 11.89, *P* = 0.0001], consistent with the expected loss of cocaine affinity at SERT shown in our previous studies [25].

### Genetic reduction of cocaine affinity for SERT fails to restore DA elevations in the striatum of DAT Val559 mice

When DA levels were assessed in the same microdialysates used for 5-HT assays, we detected significant DA elevations following cocaine administration for either wildtype or SERT Met172 mice, as expected. As predicted from prior ex vivo slice studies [19], the loss of cocaine-induced locomotor activation in DAT Val559 mice was paralleled by a loss of striatal DA elevations in these mice (Fig. 6c, d) [Two-way rmANOVA revealed a significant effect of genotype: *F*(3,21) = 4.957, *P* = 0.0093 and time: F(11,231) = 12.15, *P* < 0.0001]. Strikingly, whereas the SERT Met172 mutation restored locomotor stimulation by cocaine in DAT Val559 mice, the mutation failed to restore DA elevations (Fig. 6c, d). These findings indicate that the contribution of serotonergic mechanisms that suppress cocaine-induced locomotor activation in DAT Val559 mice are unlikely to do so via suppression of striatal vesicular DA release.

### Evidence that 5-HT_2C_R activation contributes to the loss of cocaine-induced hyperactivity in DAT Val559 mice

Though a number of 5-HT receptors have been implicated in cocaine hyperactivity [29], selective genetic [30], or pharmacological [31] attenuation of 5-HT_2C_R signaling enhances cocaine-induced hyperlocomotion whereas a 5-HT_2C_R agonist has been found to antagonize cocaine-induced locomotion [32]. Thus, we hypothesized that increased 5-HT_2C_R signaling might underlie the suppression of locomotor activation by cocaine in DAT Val559 mice. To test this idea, we assessed the ability of systemic administration of the 5-HT_2C_ receptor antagonist SDZ SER-082 (0.5 mg/kg, i.p.) to reinstate cocaine locomotor activation in DAT Val559 mice. We specifically chose a dose that fails to impact cocaine-induced locomotion in WT mice [31]. Indeed, we found that whereas at this dose, SDZ SER-082 failed to impact cocaine-induced hyperactivity in wildtype mice (Figs. 6e, g, and S6a), the drug resulted in a significant increase in horizontal activity following cocaine administration to DAT Val559 mice (Figs. 6f, h, and S6a). Two-way ANOVA revealed a significant effect of drug (*F*(3,80) = 16.04, *P* < 0.0001) and genotype (*F*(1,80) = 18.75, *P* < 0.0001). Similar results were obtained for stereotypic behaviors (Figure S6b-d) and tended to a rescue for rearing (Figure S6e-g). Together, these data support an increased ability of 5-HT_2C_R signaling to suppress locomotor activation contributing to the loss of cocaine-induced locomotor activation in DAT Val559 mice.

## Discussion

The anatomical proximity and common efferent and afferent pathways of the DA and 5-HT neurotransmitter systems position them to co-modulate similar neurobehavioral processes and for one monoamine to provide compensatory responses to changes impacting the other. For example, it has long been known that lesioning of DA neurons in neonates leads to compensatory changes in serotonergic innervation [33]. The DAT Val559 variant, identified in ADHD [14], ASD [16], and BPD [15], is associated with aberrant, transporter-mediated DA efflux in transfected cells [17] with alterations in presynaptic control of DA release and clearance and behavior, as well as basal and drug modulated behaviors, evident in vivo [19, 21, 22]. Here, we provide evidence that a functionally penetrant consequence of DAT Val559 expression is the emergence of altered serotonergic signaling, revealed through analysis of open-field locomotion after cocaine challenge. Thus, in contrast to wildtype mice, DAT Val559 mice lack a locomotor response to cocaine, with or without prior habituation to the locomotor chamber (see Materials and methods), though the ability of cocaine to bind DAT, inhibit DA uptake, and produce CPP remains intact, and the accumulation of cocaine in the brain is equivalent comparing wildtype and mutant animals. We present multiple lines of evidence that the locomotor insensitivity to cocaine of DAT Val559 mice involves the modulation of 5-HT signaling postsynaptic to presynaptic SERT as these mice exhibit normal striatal SERT levels and normal elevations of striatal 5-HT, yet display enhanced locomotor sensitivity to fluoxetine relative to wildtype mice. Indeed, the SSRI fully counteracts the locomotor stimulant actions of methylphenidate in DAT Val559 mice without significantly altering methylphenidate responses in wildtype mice. Importantly, minimization or removal of the SERT binding capacity of cocaine, either through pharmacological (i.p. administration of the cocaine analog RTI-113 or 5-HT_2C_R antagonist SDZ SER-082) or genetic (SERT Met172 mutation) means, results in significant restoration of the capacity for cocaine to induce locomotor activation in DAT Val559 mice.

### 5-HT/DA crosstalk in DAT knockout mice

Evidence for alterations of DA-dependent 5-HT plasticity was generated previously in studies of the DAT KO mouse, investigated for many years as a face-valid ADHD model due to the profound hyperactivity of these mice in a novel environment [20]. DAT KO mice also lack a locomotor response to cocaine [20] and exhibit no cocaine-stimulated increases in extracellular DA in the nucleus accumbens [34], though due to the absence of DAT expression, the natural inference is that cocaine’s interaction with DAT is the critical determinant of cocaine-induced locomotor activation. Our findings do not negate this idea, but since DATs are present and exhibit normal DA uptake in DAT Val559 mutants, and a restoration of striatal DA elevations following cocaine administration does not occur when cocaine-induced 5-HT signaling is abrogated by the SERT Met172 allele, our work reminds us that 5-HT signaling changes likely contribute to physiological and behavioral alterations incurred by perturbed DAT function. Our findings are also consistent with, and help explain, how the spontaneous hyperactivity of DAT KO mice can be rapidly reversed by drugs that increase serotonergic tone including SSRIs, 5-HT receptor agonists, and 5-HT biosynthetic precursors [35].

### Site(s) of 5-HT action in cocaine-exposed DAT Val559 mice

A number of mechanisms could drive 5-HT-dependent locomotor suppression in DAT Val559 mice including changes in serotoninergic fiber density, SERT surface expression or function, or changes in 5-HT receptor expression or sensitivity. Striatal SERT protein content is unchanged in DAT Val559 mice, and normal 5-HT elevations are observed in this region following i.p. cocaine administration, suggesting that changes in post-synaptic 5-HT signaling drive the loss of cocaine locomotor effects, though at this time we cannot rule out changes in SERT-related mechanisms in other brain regions. Indeed, tissue content of 5-HT, classically associated with increased neurotransmitter stores and/or denser 5-HT projections, is elevated in the frontal cortex of DAT Val559 mice [19], which could indicate increased serotonergic axonal density in these regions. Our data implicating the 5-HT_2C_R as a molecular entity through which SERT antagonism dampens cocaine-induced locomotor activation in DAT Val559 mice, and evidence that 5-HT_2C_R-targeted drugs can modulate cocaine-induced hyperactivity [31] suggests that key alterations arise postsynaptic to serotonergic afferents.

Our work demonstrates that the DAT Val559 variant differentially impacts two distinct dimensions of cocaine action. Whereas DAT Val559 mice lack a locomotor response to cocaine, the rewarding properties of the drug (studies here) and motivation for reward [21] appears to be intact or enhanced. Classically, these behaviors have been shown to be subserved by distinct DA circuits with the nigrostriatal pathway associated with locomotion and the mesolimbic pathway implicated in reward and locomotor sensitization [36–38]. Nigrostriatal DA neurons are strictly necessary for cocaine-induced psychomotor activation [39]. However, cocaine micro-injection into the nucleus accumbens also promotes locomotor hyperactivity [36, 40] and DA signaling in the prefrontal cortex modulates the ability of cocaine to promote psychomotor activation in mice [41]. To further complicate matters, pharmacological manipulation of 5-HT autoreceptors in the dorsal raphe changes cocaine-dependent DA elevations in the striatum [42]. Though the divergent impact of the DAT Val559 mutation on cocaine-dependent locomotion vs reward is consistent with a nigrostriatal-specific perturbation in DA homeostasis, preventing cocaine-dependent elevations in 5-HT failed to restore extracellular DA elevations in response to cocaine in the striatum of DAT Val559 mice. Though surprising, these data are consistent with the ability of tonic DA leak produced by DAT Val559 to constitutively activate presynaptic autoreceptors that then in turn block DA release, reduce TH activity, induce DAT phosphorylation, and induce DAT trafficking [19, 22]. Thus, we suspect that the suppression of DA elevations following cocaine administration to DAT Val559 mice is predominantly a direct result of DAT-mediated DA efflux and presynaptic D2 autoreceptor activation rather than serotonergic modulation. Our findings also indicate that, in DAT Val559 mice, the loss of striatal cocaine-induced DA elevations does not drive the loss of cocaine-induced hyperactivity and that 5-HT may be acting downstream or parallel to postsynaptic DA receptors or via one or more extrastriatal regions [32] to suppress locomotor activation. Indeed, 5-HT_2C_Rs, whose blockade we have shown restores the psychomotor response to cocaine in DAT Val559 mice, are expressed predominantly on GABAergic interneurons in the dorsal and ventral striatum, prefrontal cortex, and in close proximity to dopaminergic and serotonergic cell bodies in the midbrain where they have been shown to modulate a number of cocaine-mediated behaviors including acute locomotion [43], locomotor sensitization [44], self-administration [45], and place preference conditioning [46]. Additionally, one report [47] indicates that the ability of 5HT_2C_ receptors in the nucleus accumbens to reduce locomotor activation is independent of DA release, resonating with our findings. Future work will be aimed at identifying the specific circuits where altered 5-HT signaling alters cocaine responsiveness in the DAT Val559 model.

### Implications of delayed CPP extinction in DAT Val559 mice

Though they lack an acute locomotor response to cocaine, DAT Val559 mice respond to cocaine place preference conditioning, even displaying delayed extinction of this behavior. We believe delayed cocaine CPP extinction in these mice is unlikely to represent a change in reward salience or a deficit in learning, as our prior studies demonstrate an increase in reward responding and increased learning rates in rewarded tasks [21]. This behavior could, instead, represent maladaptive habit memory formation, a stimulus/response association that normally aides in minimizing cognitive effort for autonomous actions, but that can also lead to compulsive drug seeking [48]. The dorsolateral striatum has been heavily implicated in such behavior, which resonates with our recent studies demonstrating alterations in DAT phosphorylation, surface trafficking, and clearance capacity in this region that are absent in the ventral striatum of male DAT Val559 mice [22]. It is certainly possible that perturbations in 5-HT signaling also drive the delayed extinction in cocaine CPP we observe in DAT Val559 mice. In the absence of DAT, SERT expression allows for maintenance of cocaine CPP and genetic elimination of both transporters is required to abolish cocaine CPP [49]. DAT knockout mice also display delayed amphetamine CPP extinction and inhibition of 5-HT_1A_Rs prevents amphetamine CPP in DAT knockout but not wildtype mice [50]. Together these data indicate that, in the context of DA dysregulation, compensatory alterations in the 5-HT system influence discrete components of psychostimulant reward. Though we show here that 5-HT_2C_Rs drive suppression of cocaine-induced hyperlocomotion in DAT Val559 mice, global 5-HT_2C_R hyperactivity is unlikely to underlie enhanced cocaine CPP in DAT Val559 mice as 5-HT_2C_R agonists suppress cocaine-seeking behaviors [45, 46]. It is possible, however, that the actions of 5-HT_2C_Rs are confined to a specific region or circuit in DAT Val559 mice or that other 5-HT receptor signaling cascades drive alterations in cocaine reward in these mice.

### Potential clinical significance of 5-HT perturbations in models of dopamine dysfunction

Evidence of 5-HT signaling-dependent phenotypes in both the DAT KO and the DAT Val559 model reinforces the potential importance of serotonergic perturbations arising as a result of chronic hyperdopaminergia. For disorders with a strong demonstrated dopaminergic component, e.g., ADHD, these compensatory alterations in 5-HT signaling may drive a subset of behaviors such as issues with impulse control [51], a deficit also observed in DAT Val559 mice [21]. DA dysfunction has been considered primary in ADHD symptomology, in part due to the efficacy of DAT-targeted psychostimulants in ADHD treatment. However, it is important to note that DAT blockers such as methylphenidate are effective in only 60–70% of patients [52] and many children show little improvement in academic performance or social function [53, 54]. Given evidence that the development and signaling of DA and 5-HT systems are tightly linked [33], genetic or environmental changes in DA homeostasis appear to trigger parallel perturbations of 5-HT modulated processes that, together, may contribute more to the traits of ADHD and ADHD co-morbid disorders than either neurotransmitter alone. That such a focus may have therapeutic implications, we note that the SERT/NET blocker venlafaxine has shown efficacy in clinical trials for ADHD treatment comparable to psychostimulant medications, with a better side effect profile [55, 56]. Finally, individuals with a full loss of DAT function demonstrate juvenile parkinsonism/dystonia [13] a disorder that often leads to early death. With the significant serotonergic plasticity evident in our studies from a much less severe DAT perturbation, we suggest that pharmacological manipulation of specific features of serotonergic signaling may be of benefit in overcoming locomotor changes in these subjects.

## Funding and disclosure

Financial support for this work derives from the Postdoctoral Training Program in Functional Neurogenomics MH065215 (AS), an Elaine Sanders-Bush Scholar’s Award from the Vanderbilt Silvio O. Conte Center for Neuroscience Research (GLD), the Vanderbilt International Scholar Program (RG), and NIH award MH086530 (to RDB) and MH107132 (GLD). The authors declare no competing interests.

## Supplementary information

Supplementary Figures
