# Supplementary material for: Serotonin transporter inhibition and 5-HT2C receptor activation drive loss of cocaine-induced locomotor activation in DAT Val559 mice
Source: Neuropsychopharmacology. 2018 Dec 21;44(5):994–1006. doi: 10.1038/s41386-018-0301-8 (PMC6462012; doi:10.1038/s41386-018-0301-8)
Supplement: Supplementary file 1 — Supplementary Figures [file 41386_2018_301_MOESM1_ESM.docx]

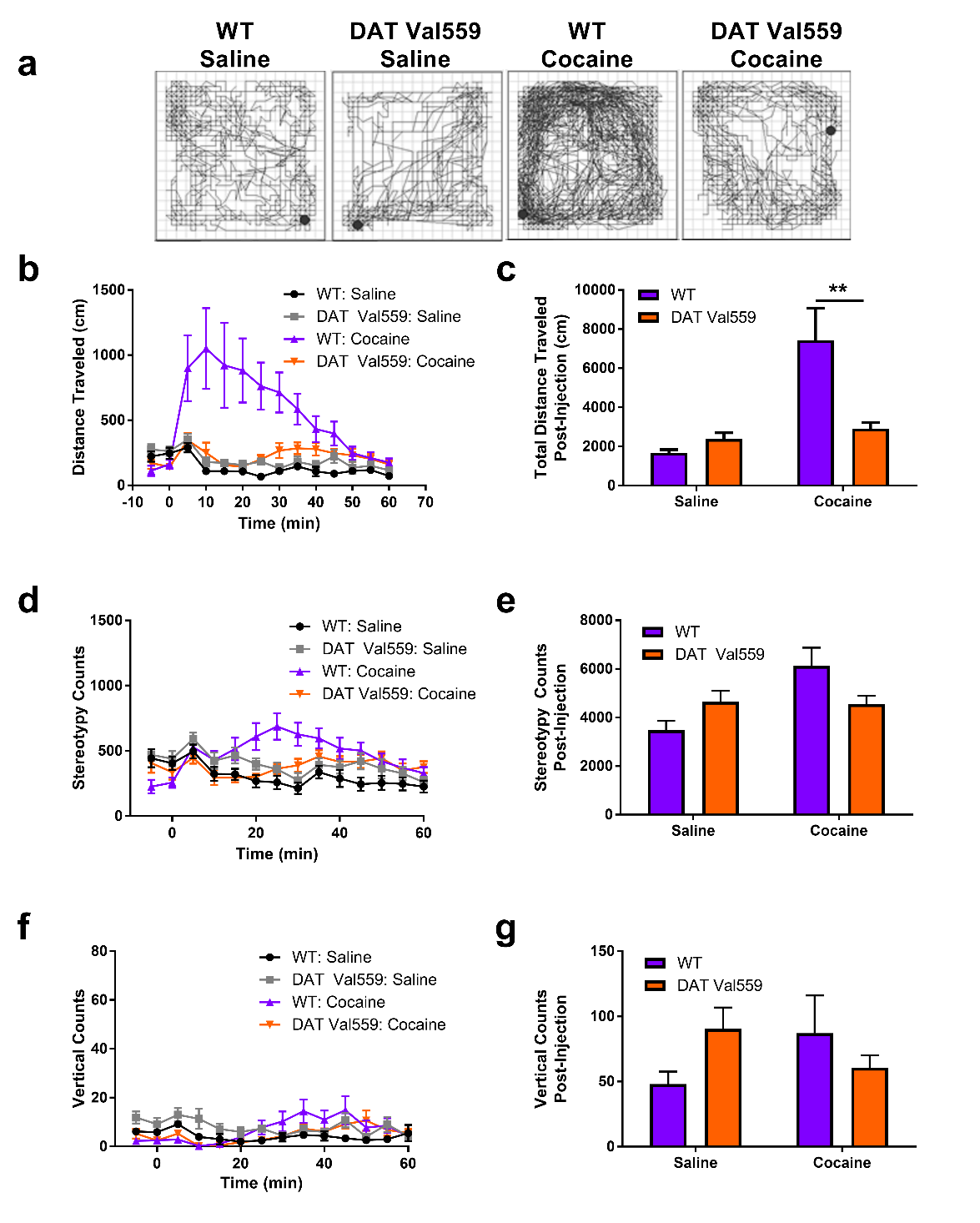


**Figure S1 – DAT Val559 mice fail to exhibit a locomotor response to high dose cocaine.** (**a**) Representative activity traces from WT (n=16) and homozygous DAT Val559 (n=16) mice injected with saline or cocaine (30 mg/kg, i.p.) and locomotor activity measured for 60 min post-injection. (**b,c**) Distance traveled, (**d,e**) stereotypy counts, and (**f,g**) vertical counts are depicted in 5 min bins or summarized as post-injections totals. ***P*<0.01 vs WT via Sidak’s post-hoc test. Data are presented as mean ± SEM.

**
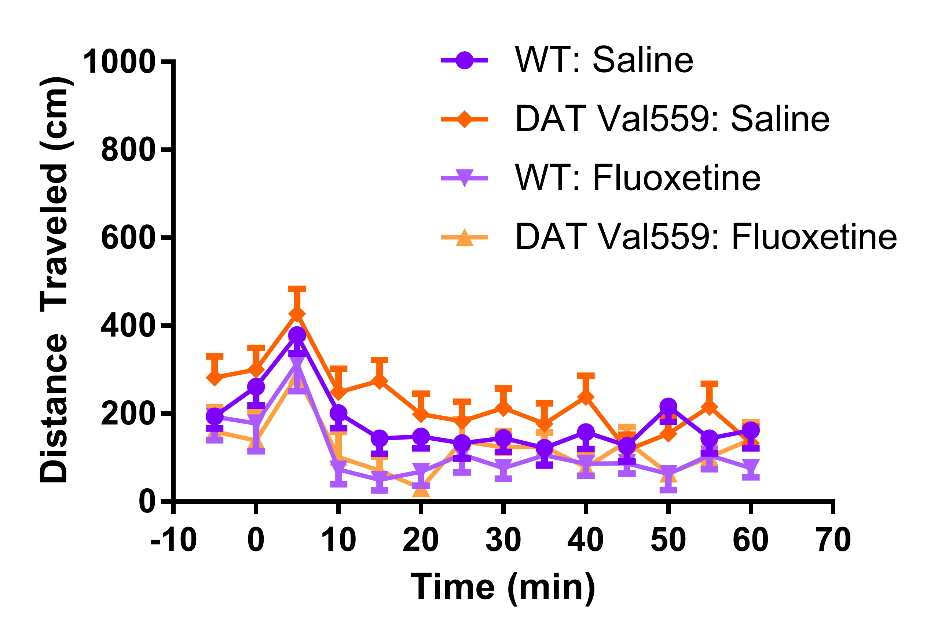
**

**Figure S2 – Fluoxetine fails to significantly impact locomotor behaviors in wildtype (WT) or DAT Val559 mice.** WT (n=12-14) and DAT Val559 (n=10-15) mice were given a single injection containing saline or fluoxetine (20 mg/kg, i.p.). Distance traveled for 60 min post-injection in 5 min bins is depicted. Data are presented as mean ± SEM.


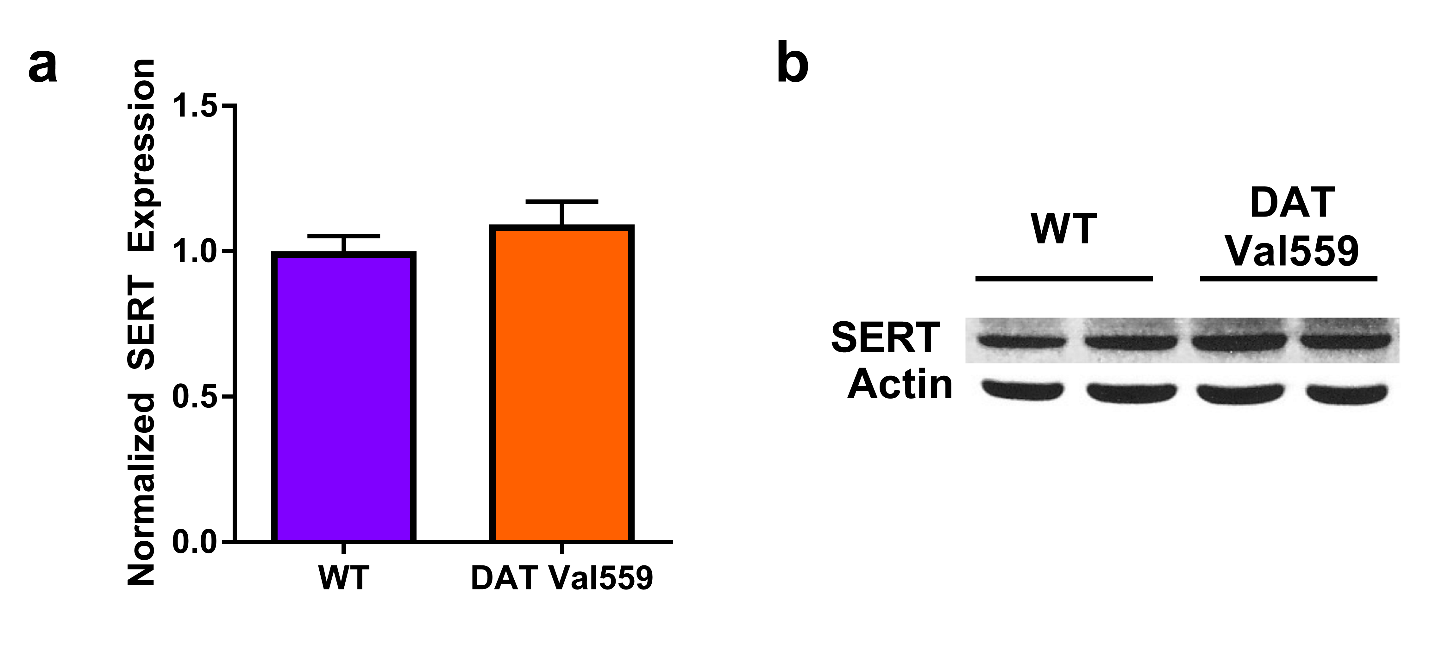


**Figure S3 – SERT protein expression is unchanged in DAT Val559 mice.** (**a**) Normalized total SERT protein expression and (**b**) representative western blots from the striatum of WT (n=16) and DAT Val559 (n=15) mice. Data were normalized to Actin loading control. Data are presented as mean ± SEM.


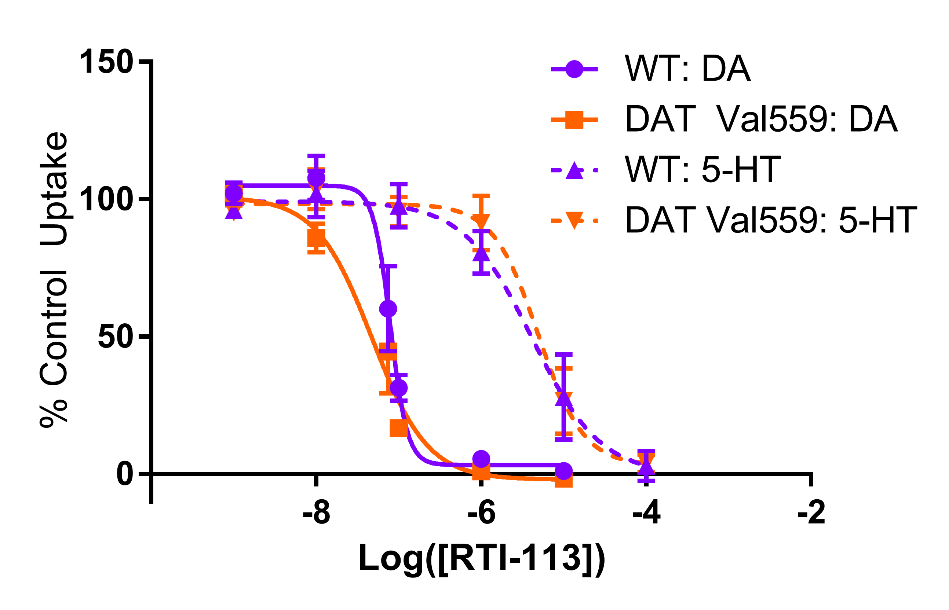


**Figure S4 – RTI-113 dependent inhibition of 5-HT and DA uptake in striatal synaptosomes isolated from wildtype (WT) and DAT Val559 mice.** Inhibition of specific DA and 5-HT uptake was assessed in striatal synaptosomes isolated from WT and DAT Val559 (n=5) exposed to increasing concentrations of RTI-113 (10^-9^ to 10^-4^ M). Nonlinear curves were fit to the data to determine IC_50_ values. For 5-HT wildtype IC_50_=4.15 ± 2.05 μM and DAT Val559, IC_50_=4.93 ± 1.77 μM. For DA wildtype, IC_50_=74.1 ± 5.5 nM and DAT Val559, IC_50_=47.8 ± 14.3 nM. Data are presented as mean ± SEM.


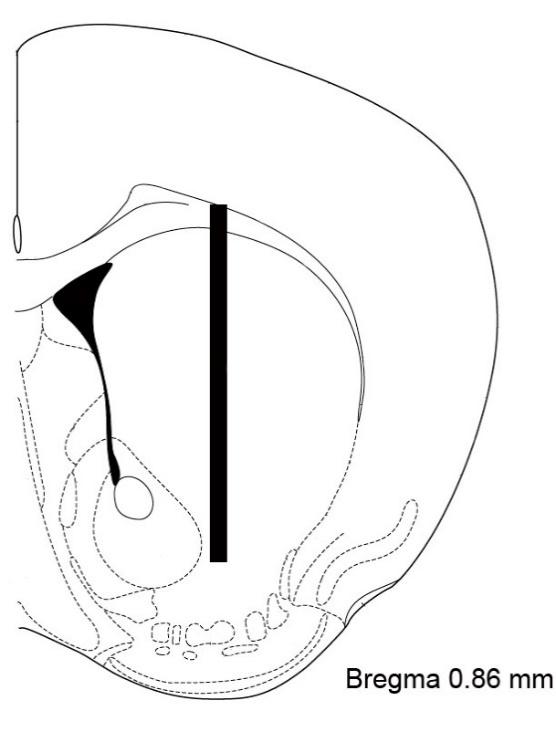


**Figure S5 – Microdialysis probe placement into the striatum.** For all microdialysis experiments probes were placed in the striatum. (-0.86 AP from bregma, ± 1.6 ML and -2.0 DV from dura).


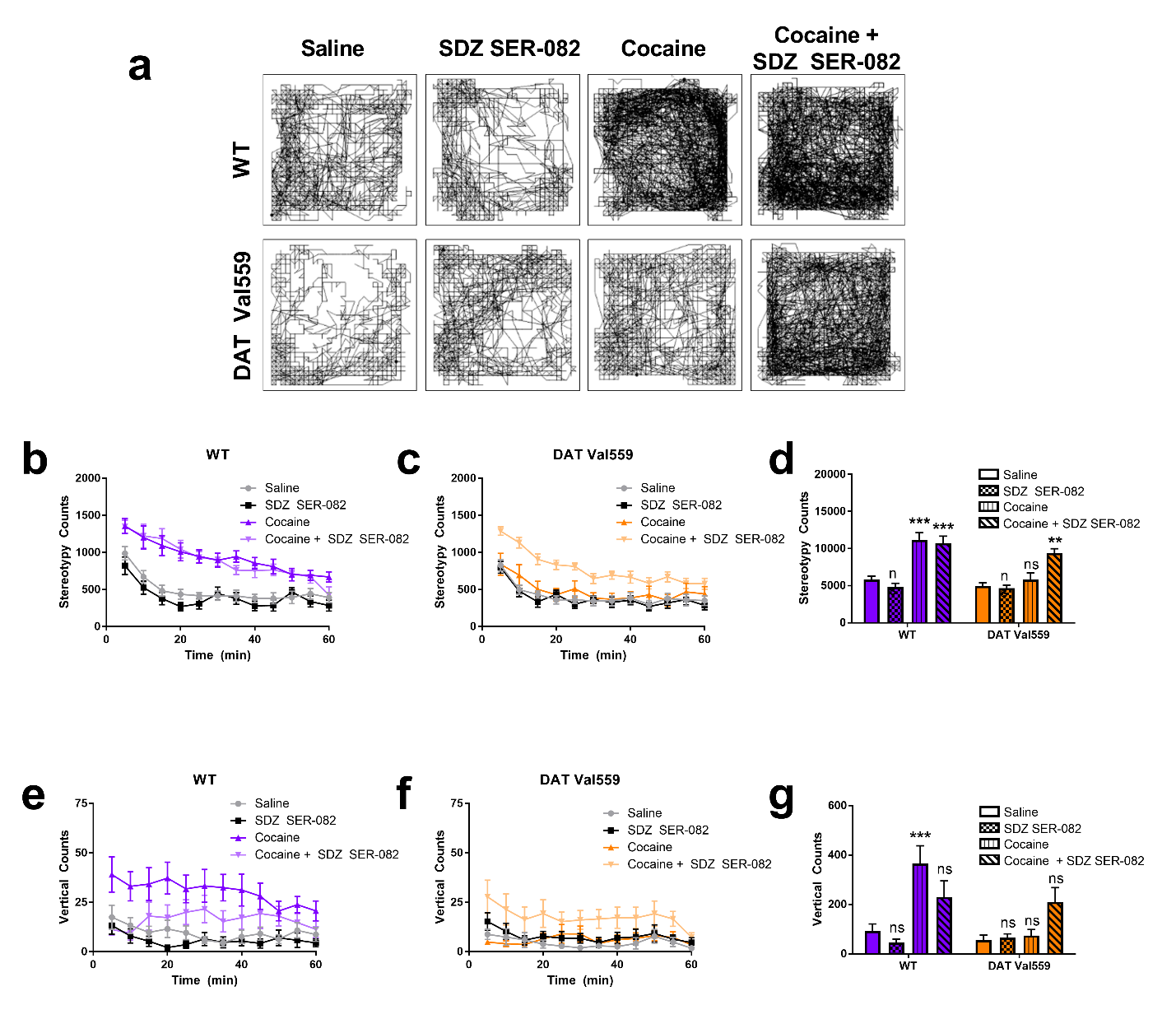


**Figure S6 – Blockade of 5-HT_2C_Rs restores cocaine-induced stereotypy and rearing in DAT Val559 mice.** (**a**) Representative activity traces of WT (n=10-12) and DAT Val559 (n=10-11) mice injected with saline or cocaine (10 mg/kg, i.p.) following a 30 min pre-exposure to saline or SDZ SER-082 (0.5 mg/kg, i.p.). (**b,c,d**) Stereotypy counts and (**e,f,g**) vertical counts are depicted in 5 min bins or summed over the 60 min recording period. Two-way ANOVA revealed a significant effect of drug (F(3,80)=28.93, *P*<0.0001) and genotype (F(1,80)=16.66, *P*<0.0001) for stereotypy and drug (F(3,80)=20.22, *P*<0.0001) and genotype (F(1,80)=5.696, *P*=0.0088) for vertical counts. ***P*<0.01, ****P*<0.001 vs saline treated control via Sidak’s post-hoc test. ns = not significantly different. Data are presented as mean ± SEM.
